# Recognition of Thyroid Ultrasound Standard Plane Images Based on Residual Network

**DOI:** 10.1155/2021/5598001

**Published:** 2021-06-02

**Authors:** Minghui Guo, Kangjian Wang, Shunlan Liu, Yongzhao Du, Peizhong Liu, Qichen Su, Guorong Lv

**Affiliations:** ^1^School of Medicine, Huaqiao University, Quanzhou 362021, China; ^2^Zhangzhou Municipal Hospital of Fujian Province, Zhangzhou 363000, China; ^3^Department of Ultrasonics, Second Affiliated Hospital of Fujian Medical University, Quanzhou 362000, China; ^4^College of Engineering, Huaqiao University, Quanzhou 362021, China; ^5^Collaborative Innovation Center for Maternal and Infant Health Service Application Technology, Quanzhou Medical College, Quanzhou 362011, China

## Abstract

Ultrasound is one of the critical methods for diagnosis and treatment in thyroid examination. In clinical application, many reasons, such as large outpatient traffic, time-consuming training of sonographers, and uneven professional level of physicians, often cause irregularities during the ultrasonic examination, leading to misdiagnosis or missed diagnosis. In order to standardize the thyroid ultrasound examination process, this paper proposes using a deep learning method based on residual network to recognize the Thyroid Ultrasound Standard Plane (TUSP). At first, referring to multiple relevant guidelines, eight TUSP were determined with the advice of clinical ultrasound experts. A total of 5,500 TUSP images of 8 categories were collected with the approval and review of the Ethics Committee and the patient's informed consent. Then, after desensitizing and filling the images, the 18-layer residual network model (ResNet-18) was trained for TUSP image recognition, and five-fold cross-validation was performed. Finally, through indicators like accuracy rate, we compared the recognition effect of other mainstream deep convolutional neural network models. Experimental results showed that ResNet-18 has the best recognition effect on TUSP images with an average accuracy rate of 91.07%. The average macro precision, average macro recall, and average macro F1-score are 91.39%, 91.34%, and 91.30%, respectively. It proves that the deep learning method based on residual network can effectively recognize TUSP images, which is expected to standardize clinical thyroid ultrasound examination and reduce misdiagnosis and missed diagnosis.

## 1. Introduction

The thyroid is one of the largest and most important endocrine organs in the human body, and it is vital to the body's metabolism. However, thyroid disease seriously threatens human health, and the incidence of thyroid cancer is increasing [1–4]. Due to its advantages of noninvasiveness, low cost, convenient examination, and good reproducibility, ultrasonography has become an essential diagnosis and treatment method for thyroid disease examination [5].

Thyroid Ultrasound Standard Plane (TUSP) is a plane for measuring thyroid parameters, an image that must be preserved in a regular thyroid ultrasound examination, and a requirement and basis for quality control of thyroid examination. Besides, TUSP can also help doctors quickly find the location of thyroid disease. In a clinical thyroid ultrasound examination, due to large outpatient traffic, time-consuming training of sonographers, and uneven professional level of physicians, doctors tend to ignore the preservation of TUSP images, and the ultrasound examination process is often not standardized. Nonstandard thyroid ultrasound examination can easily lead to missed diagnosis; then, repeated examination of patients will cause a great waste of medical resources.

One way to effectively solve these problems is to train more sonographers and carry out strict standardized training, but it requires not only investing a lot of medical funds but also spending a lot of time and energy. In recent years, with the development of artificial intelligence, especially the emergence of convolutional neural networks (CNN), computer-aided detection (CAD) technology—medical images that are automatically recognized by computer methods to assist doctors in diagnosis—has been widely used in the medical field [6, 7].

This paper aims to use TUSP images as research objects to explore a recognition method of TUSP images. By recognizing TUSP images, the sonographer can standardize the ultrasound examination process of the thyroid and reduce the misdiagnosis and missed diagnosis caused by nonstandard thyroid ultrasound examination. Besides, it is the exploration of recognition methods based on TUSP images that will help improve the efficiency of sonographer training and save medical resources.

## 2. Related Work

At present, the recognition methods widely used in ultrasound images can be divided into two types roughly. One is the image recognition and classification method based on traditional features. This method performs feature extraction, feature encoding, and feature classification on the input image to achieve image automatic recognition.

For example, in 2008, Liu et al. [8] searched for the best cross-sections of the three-dimensional ultrasound image of the heart by template matching algorithm. They achieved a high accuracy rate based on the mutual information method. In 2012, Zhang et al. [9, 10] proposed a standard plane screening method for 2D ultrasound images based on cascaded AdaBoost classifiers and local context information and proposed the concept of “intelligent ultrasound scanning”. In 2015, Huo et al. [11] designed and implemented a navigation visualization system for standard planes of transesophageal echocardiography. This system can guide doctors to find the 20 planes more and accurately and help doctors grasp the technology of getting standard planes, which facilitates it for doctors in analyzing the cases in detail to make an accurate diagnosis. In 2016, Singh et al. [12] used ten different evaluation criteria to decide the relevance of a specific feature. They obtained a classification accuracy rate of 96.6% for the 178 breast ultrasound images used in the experiment. In 2017, Khamis et al. [13] studied the automatic apical view classification method of three longitudinal scans of the echocardiograms (A2C, A4C, and ALX) for the automatic cardiac functional assessment of echocardiograms and proposed a method employing spatiotemporal feature extraction and supervised dictionary learning. Finally, the average recognition rate of the apical view of the echocardiograms achieved 95%. In 2018, Yuanet al. [14] proposed an approach based on local shape structure for detecting media-adventitia border in intravascular ultrasound (IVUS). This approach more accurately recognizes the critical points of the target border compared with other algorithms in that time and detects the whole target border successfully.

Another image recognition method is a classification method based on deep learning [15–17]. A deep network model is trained by the images to extract image features automatically, and then the image is automatically classified through the trained model.

For example, in 2015, Ni Dong's research group at Shenzhen University [18] used a pretrained neural network model to identify fetal abdominal standard plane (FASP) and used two classic neural network structures, named T-CNN and R-CNN. The network T-CNN was used to extract the ROI, and R–CNN is used to identify standard planes. The experimental results show that the accuracy of T-CNN to extract ROI reaches 90%, and the recognition rate of R-CNN reaches 82%. In 2017, Chen et al. [19] proposed a composite neural network to automatically identify fetal ultrasound standard planes: FASP, FFASP, and FFVSP from ultrasound video sequences. Experiments show that the accuracy of FASP, FFASP, and FFVSP standard slices based on ultrasound images are 90.8%, 86.7%, and 86.7%, respectively. The accuracy of FASP, FFASP, and FFVSP standard planes based on ultrasound video is 94.1%, 71.7%, and 86.4%, respectively. In 2018, Yu et al. [20, 21] proposed an automatic recognition method for fetal facial standard planes of ultrasound images based on the deep convolutional neural network framework. They achieved the recognition rate to be as high as 94.5%. In the same year, the literature [22] reported a deep learning network VP-Net used to localize multiple brain structures in three-dimensional fetal neurosonography. Based on this network, the localization results are better than other methods. In 2019, the literature [23] reported a system based on U-Net and VGG. The system locates the ultrasound standard plane first and then realizes accurate head circumference estimation based on the Obstetric Sweep Protocol (OSP) data. In 2020, to solve the problem that the field of view and orientation of the image volumes vary greatly due to the fact that clinical head CT images are obtained with different protocols, Zhang et al. [24] proposed a deep convolutional neural network called HeadLocNet. HeadLocNet is trained to classify a head CT image in terms of its content and localize landmarks to estimate a point-based registration with the same seven known landmarks. In the end, they achieved a classification accuracy of 99.5% and an average positioning error of 3.45 mm. Qu et al. [25] proposed a Deep Convolutional Neural Network (DCNN) method to automatically identify six fetal brain standard planes. Through methods such as data enhancement and transfer learning, both datasets obtained good experimental results. Wang et al. [26] proposed an attention-based feature aggregation network. This network automatically integrates multiple views of thyroid nodules obtained from a thyroid examination process and uses different views of thyroid nodules to improve the recognition effect of malignant nodules.

Since the image recognition method based on deep learning can extract the deep features of the image by constructing a deep network, the method based on deep learning has great advantages compared with traditional machine learning methods in image recognition [27]. Besides, combined with the characteristics of low contrast, low resolution, and blurred boundaries in ultrasound images, in this study, we use an 18-layer residual network [28] based on deep learning to identify TUSP.

With the approval and review of the Ethics Committee and the patient's informed consent and through cooperation with the Second Affiliated Hospital of Fujian Medical University, we have collected 5,500 TUSP images of 8 categories, manually classifying each TUSP image by the physician. After desensitizing and filling the image, we input 80% of the TUSP images into the 18-layer residual network named ResNet-18 model for training, which is used to train the model to extract the depth features of the TUSP images, and the remaining 20% of the images are used to test the recognition effect of the model on TUSP images. Finally, we conducted a comparative analysis with other mainstream network models under multiple evaluation indicators.

The main contributions of this paper are summarized as follows:

1. Referring to multiple relevant guidelines, 8 TUSP were determined to standardize clinical thyroid ultrasound examination with the advice of clinical ultrasound experts. It provides a reference for standardizing other examination processes, like fetal ultrasound.
2. A large database including 5,500 TUSP images was established to solve the clinical problems. To our best knowledge, this is the largest database of TUSP.
3. To overcome the drawback (e.g., low contrast, low resolution, and so on) from ultrasound images, an 18-layer residual network model (ResNet-18) is trained to extract the deep features of thyroid ultrasound images. To explain this method's effectiveness objectively, we compared and analyzed with a five-fold cross-validation method based on multiple evaluation indicators between ResNet-18 and other mainstream CNN models.

## 3. Methods

This study aims to standardize the thyroid ultrasound examination process to reduce missed diagnosis and other situations. Referring to multiple relevant guidelines, we define 8 TUSP in the video of the sonographer scanning the thyroid with clinical ultrasound experts' suggestions. When all 8 TUSP exist, the sonographer's examination process can be considered standard so that our task is transformed into the recognition of TUSP. To extract deep features from TUSP images, we propose using the 18-layer residual network ResNet-18 to realize the automatic classification of TUSP images.

This section will introduce the Thyroid Ultrasound Standard Plane definition and the methods we used in our study, including convolutional neural networks and ResNet networks.

### 3.1. Definition of Thyroid Ultrasound Standard Plane

**Figure 1:**
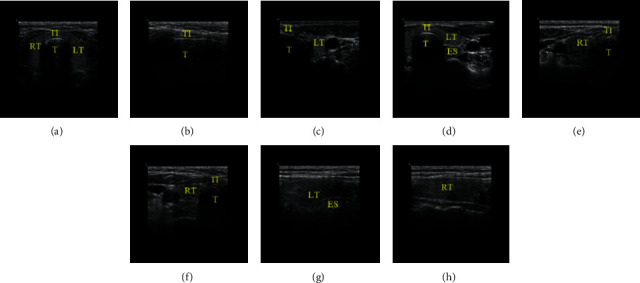
Thyroid Ultrasound Standard Plane images. (TI represents thyroid isthmus, LT and RT represent the left and right lobe of the thyroid, respectively, T represents the trachea, and ES represents esophagus). (a) TPTI, (b) LPTI, (c) UTPLT, (d) DTPLT, (e) UTPRT, (f) DTPRT, (g) LPLT, and (h) LPRT.

To observe the thyroid in detail, under the recommendations of the Clinical Ultrasound Expert Panel and various reference guides such as “*Color Atlas of Ultrasound Anatomy*” [29] and “*Ultrasound Standard Section Illustration*” [30], we define 8 TUSP during the sonographer scanning the thyroid. The 8 TUSP can roughly divide into transverse planes and longitudinal planes; they are Transverse Plane of Thyroid Isthmus (TPTI), Longitudinal Plane of Thyroid Isthmus (LPTI), Upside of the Transverse Plane of the Left lobe of Thyroid (UTPLT), Downside of the Transverse Plane of the Left lobe of Thyroid (DTPLT), Upside of the Transverse Plane of the Right lobe of Thyroid (UTPRT), Downside of the Transverse Plane of the Right lobe of Thyroid (DTPRT), Longitudinal Plane of the Left lobe of Thyroid (LPLT), and Longitudinal Plane of the Right lobe of Thyroid (LPRT), respectively. 8 categories of TUSP images are shown in Figure 1.

In Figure 1, although many planes have the same organizational structures, just like thyroid isthmus (TI) shows in TPTI, LPTI, UTPLT, DTPLT, UTPRT, and DTPRT, the focus of each plane is different. For instance, TPTI and LPTI focus on the transverse plane and longitudinal plane of TI, respectively. LPLT and LPRT focus on the longitudinal plane of the left lobe and the right of the thyroid. And UTPLT and DTPLT focus on the transverse plane of the upside and downside of the left lobe of the thyroid, respectively. UTPRT and DTPRT are similar to UTPLT and DTPLT but for the right lobe of the thyroid.

### 3.2. Convolutional Neural Network

Convolutional neural network (CNN) [31–33] is a feedforward neural network with a deep learning function designed for image recognition specifically, which has achieved great success in image recognition and detection [28, 34–37]. CNN model is usually composed of an input layer, multiple convolutional layers, pooling layers, and one (or more) fully connected layer(s).

The convolutional layer is the core of CNN, which is usually composed of multiple convolution kernels. When the image as the input signal is input into the CNN, multiple feature maps are generated through cross-correlation operations between the input signal and the first layer's convolution kernels. And these output feature maps as the input signals are input into the next layer of the CNN until the last layer. It is worth mentioning that, to reduce the number of networks' parameters and the complexity of CNN, unlike traditional artificial neural networks, CNN adopts a “weight sharing” strategy that the neurons in the same layer have the same weight. If *X*_*j*_^*l*^ represents the feature map output by the *l*-th convolutional layer and *X*_*i*_^*l*−1^ represents the feature map input by the (*l−*1)th layer, the process can be described as

(1) $$\begin{array}{c} X_{j}^{l}=f\left(X_{i}^{l-1}⊗W_{i,j}^{l}+b_{j}^{l}\right). \end{array}$$

Among them, ⊗ represents the cross-correlation operation, and *W*_*i*,*j*_^*l*^ and *b*_*j*_^*l*^ represent the weight and bias terms of the convolution kernel, respectively. Besides, the convolutional layer is usually followed by a nonlinear activation function *f*, for example, Rectified Linear Unit (*ReLu*), which is defined as *f*(*x*)=max(*x*,  0).

The pooling layer is usually designed after the convolutional layer, aiming to retain the valuable features and ignore the useless. And the output of the pooling layer is always the input data of the next layer of the CNN model. Commonly, max pooling (*max-pool*) and average pooling (*avg-pool*) are the main pooling methods. As the name implies, max pooling retains the maximum values in a specific area of the feature map, and average pooling is to retain the average values. Therefore, the pooling layer can improve the generalization ability while reducing the size of the feature map. What is more, the CNN model can be faster thanks to the reduction of parameters.

After stacking multiple convolutional layers and pooling layers, one or more fully connected layers are usually connected. The function of the fully connected layer is integrating a feature map from the previous layer into a feature vector and then use a *softmax* function to convert the feature vector into a probability distribution of the image category. Finally, the category with the highest probability is regarded as the final output of the CNN model.

### 3.3. ResNet Network Structure

There is no doubt that the depth of the network is crucial for image feature extraction. To extract deep features from TUSP images, a deep CNN is necessary to be trained. However, when the model is deeper, the degradation problem is prone to occur. As the model gets deeper and deeper, the model's performance will not increase but decrease.

**Figure 2:**
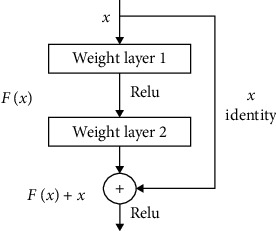
The structure of the residual block.

ResNet is a CNN model proposed by He et al. to solve the degradation problem. Residual blocks which are stacked in the model are the core of ResNet. Unlike conventional CNN stacked by multiple convolutional layers and pooling layers, each residual block is composed of 2 convolutional layers and a *short connection* [28, 38].  Figure 2 shows the structure of the residual block.

In Figure 2, *x* represents the input signal, *F*(*x*) denotes the output of the residual block before the second layer activation function. If *W*_1_ and *W*_2_ represent the weights of the first and the second layer of the residual block, respectively, *F*(*x*) can be described as *F*(*x*) = *W*_2_*f*(*W*_1_*X*) (for simplicity, the bias *b* is omitted here). In this residual block, activation function *f* uses *ReLU*, mentioned in the Convolutional Neural Network section. So, the final output of this residual block is *f*(*F*(*x*)* + x*).

Suppose the target output of the residual block is equal to the input *x*, which can be seen easily in a deep learning network. In a network with shortcut connections, we only need to optimize *F*(*x*)* + x* to *x* (or *F*(*x*) to 0). In contrast, we need to optimize *x* to *F*(*x*) = *x* in conventional CNN without shortcut connections. Therefore, shortcut connections can make the deep network easier to optimize and solve the degradation problem caused by deep networks.

**Figure 3:**
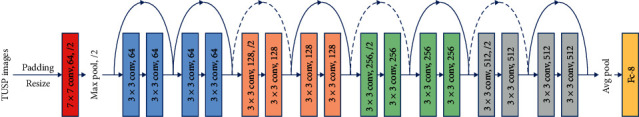
The specific structure of the ResNet-18 model.

In this study, we trained an 18-layer CNN(ResNet-18) [28] composed of one 7* *×* *7 convolutional layer, eight residual blocks, two pooling layers, and one fully connected layer to realize the automatic classification of TUSP images after padding and resizing. And each residual block is composed of two 3* *×* *3 convolutional layers.  Figure 3 shows the detail of the structure of the ResNet-18 model. And Table 1 shows the architecture of ResNet-18.

## 4. Material Collection and Experimental Process

### 4.1. TUSP Images Acquisition

The study protocol was reviewed and approved by the Ethics Committee of our institution, and informed consent was obtained from all subjects. According to the defining principle of TUSP mentioned before, we collected lots of TUSP images from the Second Affiliated Hospital of Fujian Medical University.

To ensure the quality of collected images, each TUSP image is classified by one sonographer and reviewed by two other senior sonographers. Finally, we collected 5,500 qualified and unique TUSP images; the distribution of various categories of TUSP images is shown in Table 2.

### 4.2. Image Preprocessing

**Figure 4:**
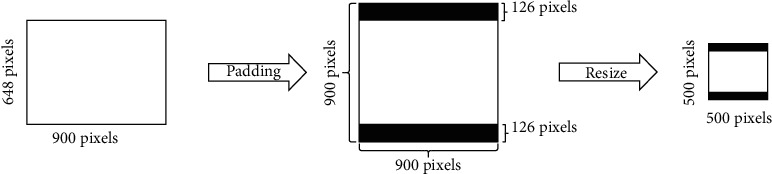
Padding and resizing of TUSP images.

TUSP images acquired from the hospital have 7 image specifications (most are 1024 × 768) due to the different models of ultrasound equipment used in hospitals. Firstly, to protect patients' privacy and uniform TUSP image size, we cropped the patient-related information. And then, we took the longest side of the image as the side length and filled the short side of the image symmetrically using 0 pixels to change the rectangular image to a square as shown in Figure 4 (Take the 900 × 648 size after clipping the privacy data as an example). Finally, the zoomed image is input into the ResNet-18 model.

### 4.3. Experimental Settings and Evaluation Indicators

This experiment is based on the Windows 10 operating system. And the specific computer hardware configuration is as follows: Intel(R) Core(TM) i7-7700, 32 GB, NVIDIA GeForce GTX-1080Ti, and video memory is 11 GB. The programming environment is Python 3.6, and the deep learning framework used in our study is TensorFlow 1.14 [39] and Keras 2.3.1.

To evaluate the recognition effect of each model objectively, we performed five-fold cross-validation of the model. The TUSP image dataset is divided into five nonoverlapping subdatasets randomly. Then the model is trained and verified five times. Four subdatasets are used to train the model (and one of these for verification), and the remaining one subset is used to test the model's performance. Moreover, each model needs to be trained and tested five times, and the subdataset used to test the model is different each time.

Besides, we applied multiple evaluation indicators to estimate the performance of the model. Precisions (*P*), recalls (*R*), and *F*1 scores (*F*1) are calculated in each category of TUSP images. The definition of *P*, *R*, and *F*1 are as follows:

(2) $$\begin{array}{c} P=\frac{\mathrm{TP}}{\mathrm{TP}+\mathrm{FP}}, \end{array}$$

(3) $$\begin{array}{c} R=\frac{\mathrm{TP}}{\mathrm{TP}+\mathrm{FN}}, \end{array}$$

(4) $$\begin{array}{c} F1=\frac{2\times P\times R}{P+R}, \end{array}$$

where TP (True Positive) represents the number of cases correctly recognized as a true category of TUSP, FP (False Positive) represents the number of cases incorrectly recognized as a true category of TUSP, TN (True Negative) represents the number of cases correctly recognized as a false category of TUSP, and FN (False Negative) represents the number of cases incorrectly recognized as a false category of TUSP.

Besides, to compare the recognition effect between the models, accuracy, macro precision (macro-*P*), macro recall (macro-*R*), and macro *F*1 score (macro-*F*1) on the test set were calculated. In our study, macro-*P*, macro-*R*, and macro-*F*1 represent the average precision, recall, and *F*1 of each type of TUSP image, respectively. The relevant formula is defined as follows:

(5) $$\begin{array}{c} \mathrm{macro}-P=\frac{1}{n}\sum_{i=1}^{n}P_{i}, \end{array}$$

(6) $$\begin{array}{c} \mathrm{macro}-R=\frac{1}{n}\sum_{i=1}^{n}R_{i}, \end{array}$$

(7) $$\begin{array}{c} \mathrm{macro}-F1=\frac{1}{n}\sum_{i=1}^{n}F1_{i}. \end{array}$$

In these equations above, *n* represents the number of TUSP image categories (equal to 8 in our experiment). *P*_*i*_, *R*_*i*_, and *F*1_*i*_ represent the precision, recall, and *F*1 score of the *i*-th categories of TUSP images, respectively.

What is more, we use the number of models' parameters to evaluate the computational cost of different models, and McNemar's test is applied to illustrate the difference between the two models with the closest performance.

## 5. Experimental Results

We trained the ResNet-18 model using the five-fold cross-validation method after TUSP images preprocessing, which was introduced before. Using the 18-layer ResNet residual network, the average recognition accuracy of TUSP images reached 91.07%, the average macro precision reached 91.39%, the average macro recall reached 91.34%, and the average macro F1 score reached 91.30%.  Table 3 shows the details.

In Table 3, ResNet-18 shows the best recognition effect on TPTI and LPTI, getting more than 98% in precision, recall, and *F*1 score. The second is identifying standard planes of UTPLT, DTPLT, UTPRT, and DTPRT, and the evaluation indicators are all above 90%. The worst recognition effect is the recognition of LPLT and LPRT. The recall, precision, and *F*1 of LPLT identification are only 78.52%, 76.80%, and 77.53%, respectively. The precision, recall, and F1 score are 81.70%, 82.72%, and 82.12%, respectively.

**Figure 5:**
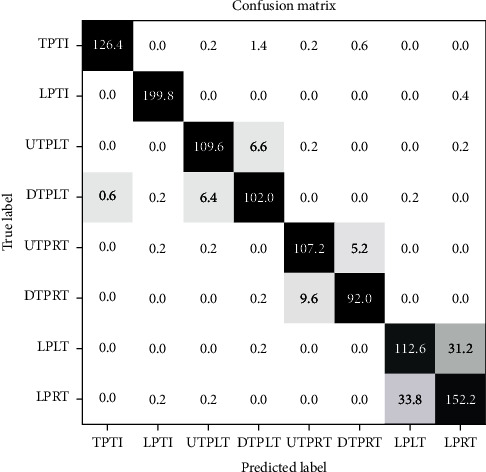
The confusion matrix of the experiment result (the values in the figure are the average of five-fold cross-validation).

Figure 5 shows the confusion matrix of the average result of the five-fold cross-validation of the ResNet-18 model. In the confusion matrix, the abscissa represents the label predicted by the model, and the ordinate represents the true label of TUSP images. The number in the figure represents the average number of TUSP images recognized by the model's five-fold cross-validation.

From the confusion matrix, we can see intuitively that the ResNet-18 can recognize most TUSP images correctly. Among them, the ResNet-18 model has the best effect on TPTI and LPTI. On average, only 0.6 images belonging to DTPLT are recognized as TPTI, and only 0.6 other TUSP images are recognized as LPTI incorrectly. The recognition effect of LPRT is the worst. On average, 33.8 LPRT images are incorrectly recognized as LPLT, and 31.2 LPLT images are recognized as LPRT incorrectly.

To compare the recognition effects on TUSP images, we trained other mainstream CNN models from scratch with random initialization. Under the same experimental conditions and same dataset, the TUSP images are scaled to the same input image size in their original paper and then inputted to ResNet-101, ResNet-152 [28], VGG16 [34], Inception V3 [35], MobileNet [36], and Xception [37]. In these models, we set the batch size to 2 due to video memory limitations. At the same time, we used the same evaluation indicators to evaluate these models. The recognition effects of the comparative experiment are shown in Table 4.

It can be seen from Table 4 that the average classification accuracy of mainstream CNN models for TUSP images has exceeded 86%. And the recognition effect of the ResNet-18 model is better than other mainstream models significantly. Its accuracy, macro-*P*, macro-*R*, and macro-*F*1, are 0.94%, 0.56%, 0.87%, and 0.83% higher than those of the second-ranked Xception model, respectively.

To describe the difference between ResNet-18 and Xception (the second-ranked model in Table 4), we applied McNemar's test with the cumulative result (not average result) of five-fold cross-validation. And the result shows that the prediction results between ResNet-18 and Xception are significantly different (*x*^2^ = 25.96, *p*-value < 0.05). Besides, from Table 5, we can find that ResNet-18 achieves better results using nearly half the parameters than Xception.

## 6. Discussion

Currently, there are many studies on CAD-based medical image recognition and classification. As for thyroid ultrasound images, most academics are paying attention to locate thyroid nodules and judge whether they are benign or malignant [26, 40–46], but little attention is paid to the standardization of thyroid ultrasound examination procedures. It is crucial of course to locate the position of thyroid nodules, but also to the process of thyroid ultrasound examinations.

In clinical, due to large outpatient traffic, time-consuming training of sonographers, and uneven professional level of physicians, doctors tend to ignore the preservation of TUSP images, and the ultrasound examination process is often not standardized. And it will lead to many problems, such as misdiagnosis and missed diagnosis.

In our study, we defined 8 TUSP in different positions of the thyroid to standardize clinical thyroid ultrasound examination, which can be referenced to standardize other examination processes (such as fetal ultrasound). Then, through cooperation with the Second Affiliated Hospital of Fujian Medical University, we collected 5,500 TUSP images in 8 categories with the approval and review of the Ethics Committee and the patient's informed consent. Besides, we trained an 18-layer residual network model (ResNet-18) to recognize TUSP images.

The experiment shows that CNN models can recognize TUSP images effectively, and the 18-layer residual network ResNet-18 gets the best. To evaluate the recognition effect of each model objectively, we use five-fold cross-validation and comparative analysis with other mainstream CNN models under multiple evaluation indicators, including accuracy, precision, recall, and F1 score. Besides, McNemar's test shows that the performance between ResNet-18 (the first-ranked model) and Xception (the second-ranked model) is significantly different. The comparative experiment shows that ResNet-18 can effectively extract features from TUSP images and the effect is better than other CNN models (as shown in Table 4).

However, there are still shortcomings in our study. First, compared with natural image datasets such as ImageNet [47], the dataset collected by our research is still small. Secondly, although CNN models get good performance in the recognition on the TUSP images, on the whole, the recognition effects on LPLT and LPRT are not very well. From Figure 5, we can see that the similarity between LPLT and LPRT is high. From Table 4, the precision, recall, and F1 score of LPLT are only 78.52%, 76.80%, and 77.53%, respectively. The precision, recall, and F1 score of LPRT are only 81.70%, 82.72%, and 82.12%, respectively.

We analyzed the reasons for the lack of experiments. Regarding the dataset problem, first of all, the acquisition of medical images is challenging and expensive because medical images involve ethics, informed consent, and others. As for the poor recognition effect on LPLT and LPRT, we believe that it is affected by at least two factors. On the one hand, the characteristics (low contrast, low resolution, blurred boundaries, artifacts, speckle noise, etc.) of ultrasound images themselves are essential factors. On the other hand, the high similarity between LPLT and LPRT(see Figure 1(g) and 1(h)) will significantly interfere with the model's recognition.

Although we have established a large database with 5500 TUSP images, and the recognition accuracy rate has reached 91.07%, there are still many challenges before clinical application. In the future, we will continue to collect TUSP images and explore a better performance model for TUSP recognition. Besides, we will develop a computer-aided diagnosis (CAD) system to standardize the examination procedures of clinicians, which can be applied in the field of clinical and sonographers' teaching and training.

## 7. Conclusion

Aiming at problems such as misdiagnosis and missed diagnosis caused by irregular thyroid ultrasound examination, we defined 8 TUSP in different positions of the thyroid. And we take TUSP as the research object to explore the method to standardize thyroid ultrasound examination procedure. Moreover, we trained a residual network-based deep learning method to recognize TUSP after preprocessing 5,500 TUSP images collected from our cooperative hospital. What is more, we compare and analyze the recognized effect from other CNN models (including ResNet models with different layer structures, VGG16, InceptionV3, MobileNet, and Xception) by the five-fold cross-validation method.

The experimental results show that CNN models can recognize TUSP images effectively. And in this study 18-layer residual network model ResNet-18 used gets the best recognition effect on TUSP images. The recognition accuracy of TUSP reached 91.07%, the macro precision reached 91.39%, the macro recall reached 91.34%, and the macro F1 score reached 91.30%. The experimental results show that the residual network can effectively recognize TUSP images, laying the foundation for the automatic standardization of thyroid ultrasound examination procedures and being expected to reduce misdiagnosis and missed diagnosis caused by irregular ultrasound examination procedures. And it is worthy of further exploration. What is more, it may become an effective way to save medical resources and speed up the training of sonographers.

## Figures and Tables

**Table 1:** Architecture of ResNet-18.

Block	Layers	Output size
Input	Input layer	500 × 500 × 1
Conv 1	7* *×* *7 conv	250 × 250 × 64
Pooling	2* *×* *2 max pooling	125 × 125 × 64
Residual block 1	3* *×* *3 conv	125 × 125 × 64
	3* *×* *3 conv	
Residual block 2	3* *×* *3 conv	125 × 125 × 64
	3* *×* *3 conv	
Residual block 3	3* *×* *3 conv	63 × 63 × 128
	3* *×* *3 conv	
Residual block 4	3* *×* *3 conv	63 × 63 × 128
	3* *×* *3 conv	
Residual block 5	3* *×* *3 conv	32 × 32 × 256
	3* *×* *3 conv	
Residual block 6	3* *×* *3 conv	32 × 32 × 256
	3* *×* *3 conv	
Residual block 7	3* *×* *3 conv	16 × 16 × 512
	3* *×* *3 conv	
Residual block 8	3* *×* *3 conv	16 × 16 × 512
	3 × 3 conv	
Avg pooling	16 × 16 avg pooling	1 × 1 × 512
FC layer	FC softmax	1 × 1 × 8

**Table 2:** Distribution of 8 categories of TUSP images.

Types of TUSP	Number	Types of TUSP	Number
TPTI	635	UTPRT	586
LPTI	1002	DTPRT	489
UTPLT	583	LPLT	733
DTPLT	552	LPRT	920
Sum	5500		

**Table 3:** The precision, recall, and F1 score of various categories in the test set.

Types of TUSP	Precision	Recall	*F*1 score
TPTI	0.9815	0.9953	0.9883
LPTI	0.9980	0.9970	0.9975
UTPLT	0.9410	0.9399	0.9401
DTPLT	0.9328	0.9241	0.9277
UTPRT	0.9512	0.9146	0.9322
DTPRT	0.9046	0.9408	0.9218
LPLT	0.7852	0.7680	0.7753
LPRT	0.8170	0.8272	0.8212
Macro average	**0.9139**	**0.9134**	**0.9130**
Accuracy	**0.9107**		

The values in the table are the average of five-fold cross-validation.

**Table 4:** Recognition effects of different CNN models on TUSP images.

Models		TPTI	LPTI	UTPLT	DTPLT	UTPRT	DTPRT	LPLT	LPRT	Macro average	Accuracy
ResNet-18	*P*	0.9815	**0.9980**	**0.9410**	0.9328	**0.9512**	0.9046	**0.7852**	**0.8170**	**0.9139**	**0.9107**
	*R*	**0.9953**	0.9970	0.9399	0.9241	0.9146	**0.9408**	**0.7680**	0.8272	**0.9134**	
	*F*1	0.9883	**0.9975**	0.9401	0.9277	**0.9322**	0.9218	**0.7753**	**0.8212**	**0.9130**	

ResNet-50	*P*	0.9845	0.9832	0.9026	0.8956	0.9179	0.8792	0.6884	0.7812	0.8791	0.8744
	*R*	0.9843	0.9890	0.9004	0.8842	0.8909	0.9100	0.7329	0.7348	0.8783	
	*F*1	0.9843	0.9861	0.9006	0.8890	0.9030	0.8929	0.7078	0.7558	0.8775	

ResNet-101	*P*	0.9922	0.9902	0.9117	0.9281	0.9040	0.8964	0.7492	0.7242	0.8870	0.8795
	*R*	0.9906	0.9910	0.9296	0.9041	0.9061	0.8876	0.6127	0.8261	0.8810	
	*F*1	0.9913	0.9906	0.9203	0.9156	0.9048	0.8909	0.6710	0.7703	0.8818	

VGG16	*P*	0.9937	0.9851	0.9328	0.8929	0.9039	0.8798	0.7190	0.7461	0.8817	0.8762
	*R*	0.9874	0.9900	0.8971	0.9221	0.8911	0.8813	0.6768	0.7815	0.8784	
	*F*1	0.9905	0.9875	0.9132	0.9057	0.8956	0.8784	0.6963	0.7626	0.8787	

ResNet-152	*P*	**0.9938**	0.9813	0.9247	0.9028	0.8826	0.8538	0.7800	0.7024	0.8777	08634
	*R*	0.9858	0.9910	0.9057	**0.9258**	0.8768	0.8569	0.5289	**0.8370**	0.8635	
	*F*1	0.9897	0.9861	0.9143	0.9128	0.8773	0.8507	0.6042	0.7556	0.8613	

InceptionV3	*P*	0.9907	0.9911	0.9359	0.9341	0.9220	0.8960	0.7430	0.7926	0.9007	0.8962
	*R*	0.9858	0.9920	0.9398	0.9313	0.9098	0.9140	0.7407	0.7870	0.9000	
	*F*1	0.9881	0.9915	0.9374	**0.9320**	0.9150	0.9038	0.7398	0.7882	0.8995	

MobileNet	*P*	0.9892	0.9921	0.9341	0.9294	0.9162	**0.9118**	0.7490	0.8028	0.9031	0.8986
	*R*	0.9937	**0.9960**	0.9347	0.9223	**0.9199**	0.8936	0.7613	0.7880	0.9012	
	*F*1	**0.9914**	0.9940	0.9340	0.9254	0.9174	0.9018	0.7528	0.7932	0.9012	

Xception	*P*	0.9844	0.9913	0.9298	**0.9605**	0.9504	0.9054	0.7634	0.7812	0.9083	0.9013
	*R*	0.9890	0.9900	**0.9639**	0.9061	0.9148	0.9406	0.7015	0.8315	0.9047	
	*F*1	0.9867	0.9906	**0.9461**	0.9319	0.9318	**0.9222**	0.7258	0.8030	0.9047	

The values in the table are the average of five-fold cross-validation.

**Table 5:** The computational cost of different CNN models.

CNN models	Trainable parameters	Nontrainable parameters	Total parameters
ResNet18	11,177,352	7,808	11,185,160
ResNet50	23,544,712	53,120	23,597,832
ResNet101	42,562,952	105,344	42,668,296
ResNet152	58,229,640	151,424	58,381,064
VGG16	134,292,168	0	134,292,168
InceptionV3	21,784,168	34,432	21,818,600
MobileNet	3,214,600	21,888	3,236,488
Xception	20,822,768	54,528	20,877,296

## Data Availability

The Thyroid Ultrasound Standard Plane images data used to support the findings of this study were supplied by the Second Affiliated Hospital of Fujian Medical University in Fujian, China, under license and so cannot be made freely available.
